# Immobilization of Bi_2_WO_6_ on Polymer Membranes for Photocatalytic Removal of Micropollutants from Water – A Stable and Visible Light Active Alternative

**DOI:** 10.1002/gch2.202300198

**Published:** 2024-02-28

**Authors:** Kristina Fischer, Amira Abdul Latif, Jan Griebel, Andrea Prager, Omid Shayestehpour, Stefan Zahn, Agnes Schulze

**Affiliations:** ^1^ Leibniz Institute of Surface Engineering (IOM) Permoserstr. 15 04318 Leipzig Germany

**Keywords:** bismuth tungstate, crystallinity, microfiltration membrane, photocatalysis

## Abstract

In this work, bismuth tungstate Bi_2_WO_6_ is immobilized on polymer membranes to photocatalytically remove micropollutants from water as an alternative to titanium dioxide TiO_2_. A synthesis method for Bi_2_WO_6_ preparation and its immobilization on a polymer membrane is developed. Bi_2_WO_6_ is characterized using X‐ray diffraction and UV–vis reflectance spectroscopy, while the membrane undergoes analysis through scanning electron microscopy, X‐ray photoelectron spectroscopy, and degradation experiments. The density of states calculations for TiO_2_ and Bi_2_WO_6_, along with PVDF reactions with potential reactive species, are investigated by density functional theory. The generation of hydroxyl radicals OH^•^ is investigated via the reaction of coumarin to umbelliferone via fluorescence probe detection and electron paramagnetic resonance. Increasing reactant concentration enhances Bi_2_WO_6_ crystallinity. Under UV light at pH 7 and 11, the Bi_2_WO_6_ membrane completely degrades propranolol in 3 and 1 h, respectively, remaining stable and reusable for over 10 cycles (30 h). Active under visible light with a bandgap of 2.91 eV, the Bi_2_WO_6_ membrane demonstrates superior stability compared to a TiO_2_ membrane during a 7‐day exposure to UV light as Bi_2_WO_6_ does not generate OH^•^ radicals. The Bi_2_WO_6_ membrane is an alternative for water pollutant degradation due to its visible light activity and long‐term stability.

## Graphical abstract

The photocatalyst bismuth tungstate Bi_2_WO_6_ is immobilized on polyethersulfone and polyvinylidene fluoride microfiltration membranes to remove micropollutants from water as a visible light‐active and stable alternative to titanium dioxide TiO_2_. Bi_2_WO_6_ does not degrade the supporting polymer membrane and degrades propranolol with UVA and visible light in an efficient way. Compared to TiO_2_, Bi_2_WO_6_ shows a similar high photocatalytic activity.

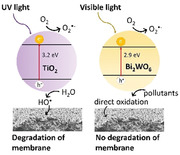

## 1 Introduction

Wastewater is increasingly contaminated with micropollutants such as cleaning agents, insecticides, herbicides, and pharmaceuticals.^[^ ^1^ ^]^ Micropollutants are already present in drinking water at trace levels (ng L^1^–µg L^1^),^[^ ^2^ ^]^ thus acute toxic effects are not of concern. But the endocrine‐disrupting effects of some micropollutants, in addition to sex changes in animals and humans, have a drastic effect on the central nervous system^[^ ^3^ ^]^; costing hundreds of billions of Euros per year in the European Union.^[^ ^4^ ^]^ Also, the toxicity of mixtures and long‐term exposure can lead to chronic effects even at very low concentrations^[^ ^5^ ^]^ while antibiotic resistance of microorganisms and accumulation in soils, plants, and animals is an increasing problem.^[^ ^6^ ^]^ Current drinking water treatment plants (sedimentation, filtration, and disinfection) were not designed to and cannot fully remove micropollutants.^[^ ^2^, ^5^, ^7^ ^]^ Among the more established methods to remove micropollutants from water is ozonation^[^ ^8^ ^]^ often the first choice next to activated carbon^[^ ^7^, ^9^ ^]^ as it is well‐known and technologically advanced. But ozone has some major drawbacks as it reacts with many metals, is energy intensive to produce (expensive),^[^ ^10^ ^]^ and is toxic. Furthermore, ozone reacts with bromide to form bromate,^[^ ^11^ ^]^ which is possibly carcinogenic for humans. Thus, operators must either control the pH or avoid the use of ozone if the water is rich in bromide salts. Activated carbon adsorbs many different micropollutants^[^ ^12^ ^]^ and a removal of up to 99% can be achieved^[^ ^13^ ^]^ but adsorption capability differs with the chemical nature of the compound and is pH dependent.^[^ ^14^ ^]^ Additionally, activated carbon just transfers micropollutants from one phase to another, leaving behind toxic waste that is expensive to treat and regenerate. Membrane systems have been used for water treatment, with pressure‐driven membrane processes being by far the most commonly used membrane techniques in water treatment. High‐pressure membrane processes like reverse osmosis and nanofiltration are effective methods to remove micropollutants by 98–99%.^[^ ^15^ ^]^ However, the challenge remains with high energy requirements due to the applied pressure,^[^ ^16^ ^]^ incomplete removal because of diffusion through the polyamide layer,^[^ ^17^ ^]^ and resupply of salts to gain drinking water quality.

**Figure 1:**
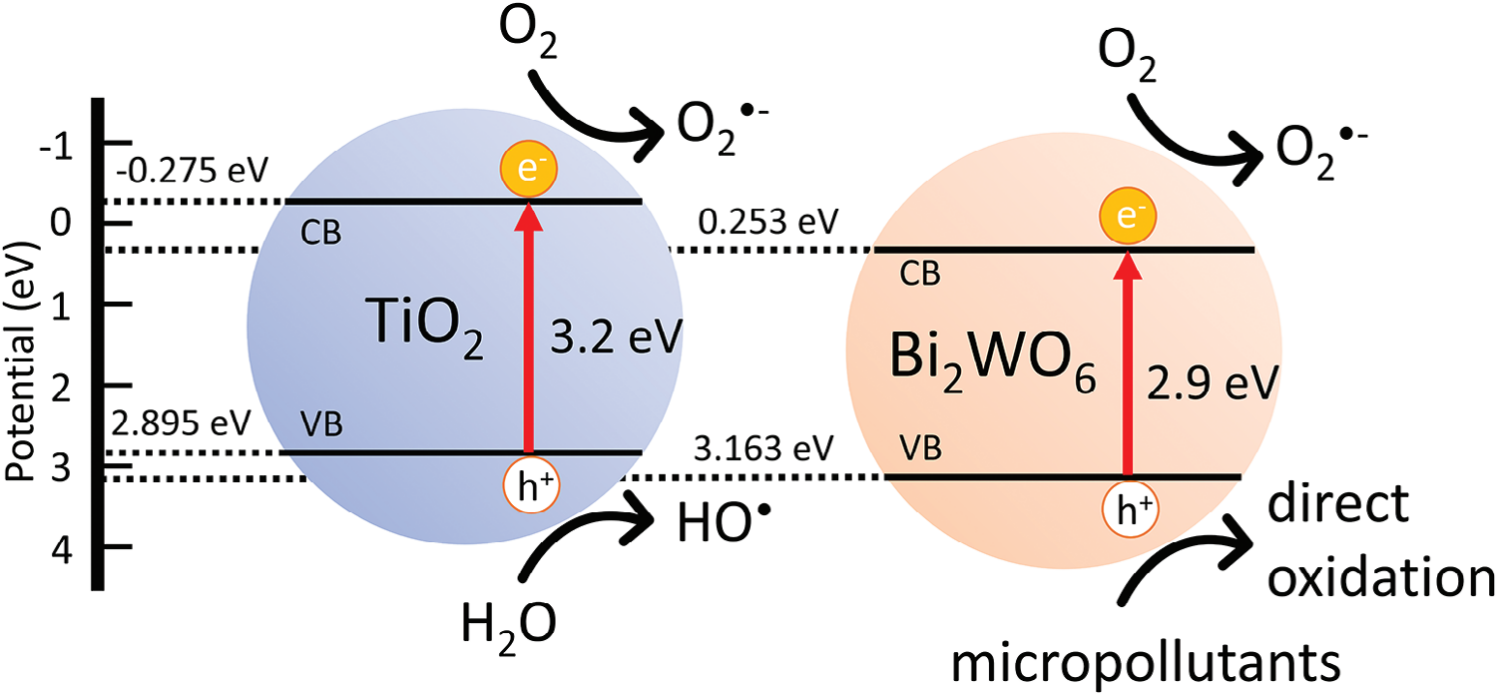
Scheme of energy bands of titanium dioxide TiO_2_ and bismuth tungstate Bi_2_WO_6_ and possible reactions to form ROS (adapted from Liu et al.^[^ ^18a^ ^]^). VB: valence band; CB: conduction band

Advanced oxidation processes (e.g., hydrogen peroxide, or titanium dioxide TiO_2_ with UVA light) efficiently clean water from organic micropollutants by completely mineralizing the pollutants into carbon dioxide, water, and mineral acids. The degradation is based on the formation of reactive oxygen species (ROS, hydroxyl radicals OH^•^, superoxide radicals O_2_^•−^), which unselectively oxidize organic compounds in water (**Figure** **1**).^[^ ^18^ ^]^ Redox reactions with water and dissolved oxygen generate highly reactive OH^•^ and O_2_^•−^ radicals. Electron‐hole recombination is not desired.

TiO_2_ has many advantages over other advanced oxidation processes and heterogeneous photocatalysts. TiO_2_ is photostable, inexpensive, chemically stable, insoluble, reusable, and non‐toxic. TiO_2_ is well‐known and studied for decades. The combination of the photocatalyst TiO_2_ with a polymer membrane system has been proven to be effective in removing micropollutants from water via ultraviolet (315–400 nm, UVA) light illumination.^[^ ^19^ ^]^ The porous membrane acts as a substrate for the photocatalyst and can be applied in a flow‐through configuration with high reaction rates. The high removal rates can be attributed to the membrane's nanoscale dimensions, which offer an extensive surface area and promote direct interaction between radicals and micropollutants.^[^ ^19k^ ^]^ However, there are two major drawbacks in using TiO_2_ as a photocatalyst in combination with a polymer membrane. First, TiO_2_ is only active under UVA light with a bandgap larger than 3.0 eV (see Figure 1). Thus, its suitability for sunlight applications is limited as less than 4% of the solar spectrum is only used.^[^ ^20^ ^]^ Doping of TiO_2_ with anions and metal cations has been evaluated to use TiO_2_ as a visible light photocatalyst.^[^ ^21^ ^]^ Unfortunately, the introduced states can act as recombination centers at the same time and thermal instability of the doped TiO_2_ diminishes their performances.^[^ ^22^ ^]^ Secondly, the generated OH^•^ radicals destroy the polymer membrane in long time usage.^[^ ^23^ ^]^ The stability of the used polymers against UV light and oxidative stress depends on the chemical stability of the bonds in the polymer.^[^ ^23a^ ^]^ Fluor‐containing polymers like polyvinylidene fluoride PVDF and polytetrafluoroethylene PTFE are the most stable ones.^[^ ^23^ ^]^

Bismuth tungstate Bi_2_WO_6_ can be an answer to the above‐addressed drawbacks of TiO_2_. It is active in visible light with a bandgap at 2.9 eV^[^ ^24^ ^]^ (Figure 1) and, unlike TiO_2,_ does not form OH^•^ radicals that attack the polymer. Hydroxyl radicals are very reactive and react with polymers via hydrogen abstraction, addition, and electron transfer.^[^ ^25^ ^]^ Bi_2_WO_6_ degrades organic molecules via O_2_^•−^ radicals or via direct oxidation by the hole h^+^. The generation of ROS by Bi_2_WO_6_ is dissimilar compared to TiO_2_ as the bands are located at different energies (see Figure 1). From the theoretical point, the formation of OH^•^ is nearly impossible. The valence band in Bi_2_WO_6_ is formed by Bi^3+^ and holes generated by photoexcitation are regarded as Bi^5+^ or Bi^4+^. The standard redox potential of Bi^V^/Bi^III^ (E° = +1.59 V at pH 0) is lower than that of OH^•^/OH^−^ (+1.99 V), thus the photogenerated hole on the surface of Bi_2_WO_6_ cannot react with OH^−^/H_2_O to form OH^•^ radicals.^[^ ^26^ ^]^ The narrow bandgap can be explained by [Bi_2_O_2_] and perovskite‐type layers which promote good electron conductivity and visible light absorption. Bi_2_WO_6_ is convenient due to its low cost, nontoxicity, modifiable morphology, environmental friendliness, and high thermal and (photo)chemical stability.^[^ ^24^, ^27^ ^]^ Bi_2_WO_6_, being among the most basic Aurivillius oxides, exhibits an orthorhombic structure. This structure consists of layers composed of [Bi_2_O_2_]^2+^ connected to [WO_4_]^2−^ layers through shared corners.^[^ ^28^ ^]^ The distinctive sandwich‐like arrangement of Bi_2_WO_6_ creates an inherent electric field between these layers, which proves advantageous for the efficient separation of photogenerated electrons and holes.^[^ ^27^ ^]^ Bi_2_WO_6_ has been synthesized by diverse methods like sol‐gel, co‐precipitation, molten salt, solvothermal method, and hydrothermal process.^[^ ^29^ ^]^ Solvothermal and hydrothermal synthesis generally applies the salts bismuth (III) nitrate Bi(NO_3_)_2_ and sodium tungstate Na_2_WO_4_.^[^ ^24^ ^]^ Research on Bi_2_WO_6_ is an ongoing process to maximize their photocatalytic activity. Composite fabrication^[^ ^27^, ^30^ ^]^ including S‐scheme^[^ ^31^ ^]^ and Z‐scheme^[^ ^32^ ^]^ heterojunction and carbon‐based composites^[^ ^33^ ^]^ has been applied to increase the separation of photogenerated carriers. With only a single component in the photocatalytic system, the likelihood of charge recombination is high. The development of composite photocatalysts is widely recognized as a popular and effective strategy for enhancing the separation of charge carriers. Defect engineering including oxygen vacancies^[^ ^34^ ^]^ has the capacity to modify the electronic characteristics of materials and the interplay among atoms, including factors like bond energy, binding energy, electronegativity, and charge density distribution.^[^ ^27^ ^]^ These alterations can subsequently influence the dielectric properties, optical characteristics, and thermodynamic properties of the materials.^[^ ^27^ ^]^ Doping^[^ ^33^ ^]^ has become also for Bi_2_WO_6_ a prominent tool to modify its energy band structure, broaden the spectrum of light absorption, and enhance the efficiency of separating photogenerated carriers.

Here, the synthesis of highly active Bi_2_WO_6_ and its immobilization on polymer microfiltration membranes is presented as a possible alternative to TiO_2_ to overcome its disadvantages of being not active under visible light and lacking long‐term stability due to OH^•^ radical attack of the supporting membrane polymer. A synthesis route of Bi_2_WO_6_ is examined to gain highly photocatalytic activity by differing the concentration of reactant salts. The crystallinity and bandgap were determined via X‐ray diffraction (XRD) and Ultraviolet–visible (UV–vis) reflectance spectroscopy, respectively. Density functional theory (DFT) was employed to examine calculations of the density of states for TiO_2_ and Bi_2_WO_6_, as well as the reactions of PVDF with potential reactive species. An immobilization method was developed to gain a thorough immobilization of Bi_2_WO_6_ on polymer membrane surfaces. The Bi_2_WO_6_ polymer membrane was characterized by scanning electron microscopy (SEM), X‐ray photoelectron spectroscopy (XPS), and degradation experiments of methylene blue and propranolol in batch mode at different pH values and for 10 cycles under UVA and visible light.

## 2 Results and Discussion

### 2.1 Influence of Reactant Salt Concentration

The effect of reactant salt (sodium tungstate dihydrate Na_2_WO_4_*2 H_2_O and bismuth (III) nitrate pentahydrate Bi(NO_3_)_3_*5 H_2_O) concentration during synthesis on the crystallinity and photocatalytic activity was examined.

**Figure 2:**
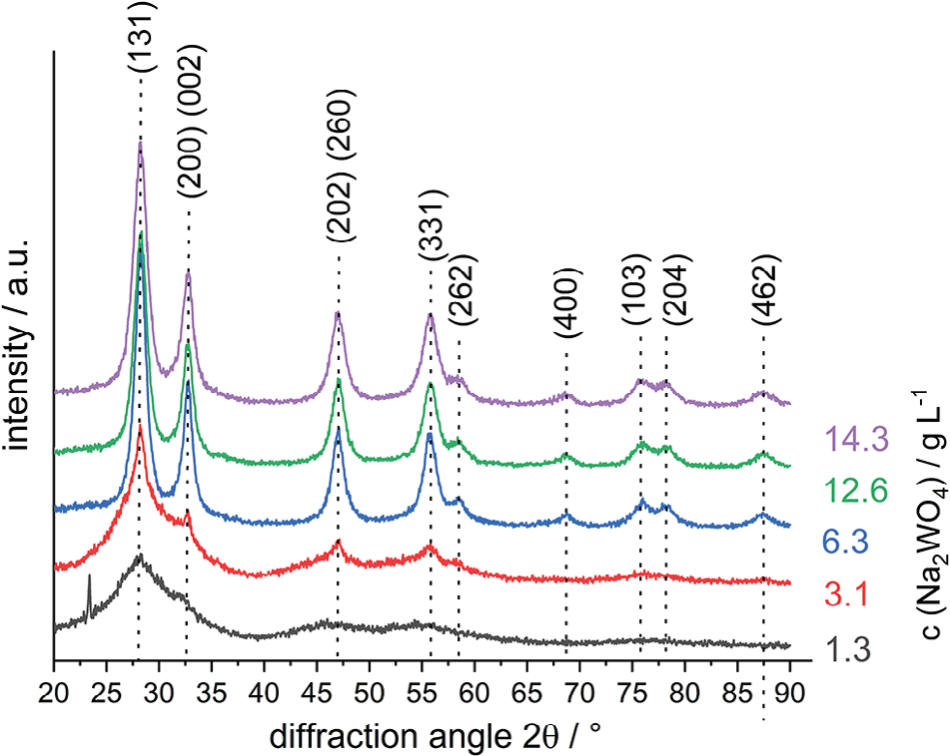
XRD pattern of Bi_2_WO_6_ powder synthesized at different concentrations of reactant salts (here stated as the concentration of Na_2_WO_4_*2 H_2_O). The reactant salts Na_2_WO_4_*H_2_O and Bi(NO_3_)_3_*5H_2_O were applied at a ratio of 1:2.9, respectively.

The intensity and form of the reflexes in the XRD diffractogram (see **Figure** **2**) change with varying concentrations of the reactant salts (ratio of Na_2_WO_4_*2 H_2_O to Bi(NO_3_)_3_*5 H_2_O was kept always at 1:2.9, thus only the concentration of Na_2_WO_4_*2 H_2_O is given further on). All the diffraction reflexes can be indexed to orthorhombic Bi_2_WO_6_ and no traces of other impurity phases are detected. The peaks become narrower, and the intensity increases with rising concentration of reactant salts until a concentration of 6.3 g L^−1^ of Na_2_WO_4_*2 H_2_O is reached. No further intensity increase can be detected between a concentration of 6.3 and 14.3 g L^−1^ of Na_2_WO_4_*2 H_2_O. The high intensity and narrow peaks of the Bi_2_WO_6_ powder synthesized with a concentration of 6.3 g L^−1^ of Na_2_WO_4_*2 H_2_O and above indicate a high crystallinity.

Improved crystallinity is sought for photocatalysts to increase the activity and degradation of organic materials.^[^ ^35^ ^]^ The diffusion time of the light‐generated carriers (e^−^ and h^+^) is influenced by the crystallinity. With a high crystallinity and diffusion time the recombination of e^−^ and h^+^ is reduced and more carriers are allocated at the surface to react with the surrounding medium (water, organic molecules) to oxidate directly or generate radicals. The diffusion time (τ) is described as

(1) $$\tau \;=k_{D}\;\frac{r_{\mathrm{CZ}}^{2}}{\pi D}$$

where *k*_D_, *r*_CZ_, and *D* are the coefficient for the interaction, the crystallite size, and the diffusion coefficient of a carrier, respectively.^[^ ^36^ ^]^ The *k*_D_ can be correlated to the efficiency of the catalyst to absorb light and the type of energy gap.^[^ ^35a^ ^]^ The density of defects is also decreased with increased crystallinity. Defects act as recombination centers for the e^−^ and h^+^ pair,^[^ ^37^ ^]^ thus photocatalytic activity is directly correlated.

Bi_2_WO_6_ synthesized with different concentrations of reactant salts (Na_2_WO_4_*2 H_2_O and Bi(NO_3_)_3_*5 H_2_O) was immobilized on polyethersulfone (PES) membranes and the amount of bismuth on the membrane surface was analyzed (amount of Bi in atom‐% examined via XPS, Figure S1, Supporting Information). Only a very small amount of Bi is found on the surface for Bi_2_WO_6_ synthesized with a reactant salt (Na_2_WO_4_*2 H_2_O) concentration of 3.1 g L^−1^ and 6.3 g L^−1^, compared to an reactant salt concentration of 1.3, 12.6 and 14.3 g L^‐1^ (e.g., amount of Bi of (0.09 ± 0.05)% for 6.3 g L^−1^ of Na_2_WO_4_*2 H_2_O compared to an amount of Bi of (0.37 ± 0.05)% for 14.3 g L^−1^ of Na_2_WO_4_*2 H_2_O).

**Figure 3:**
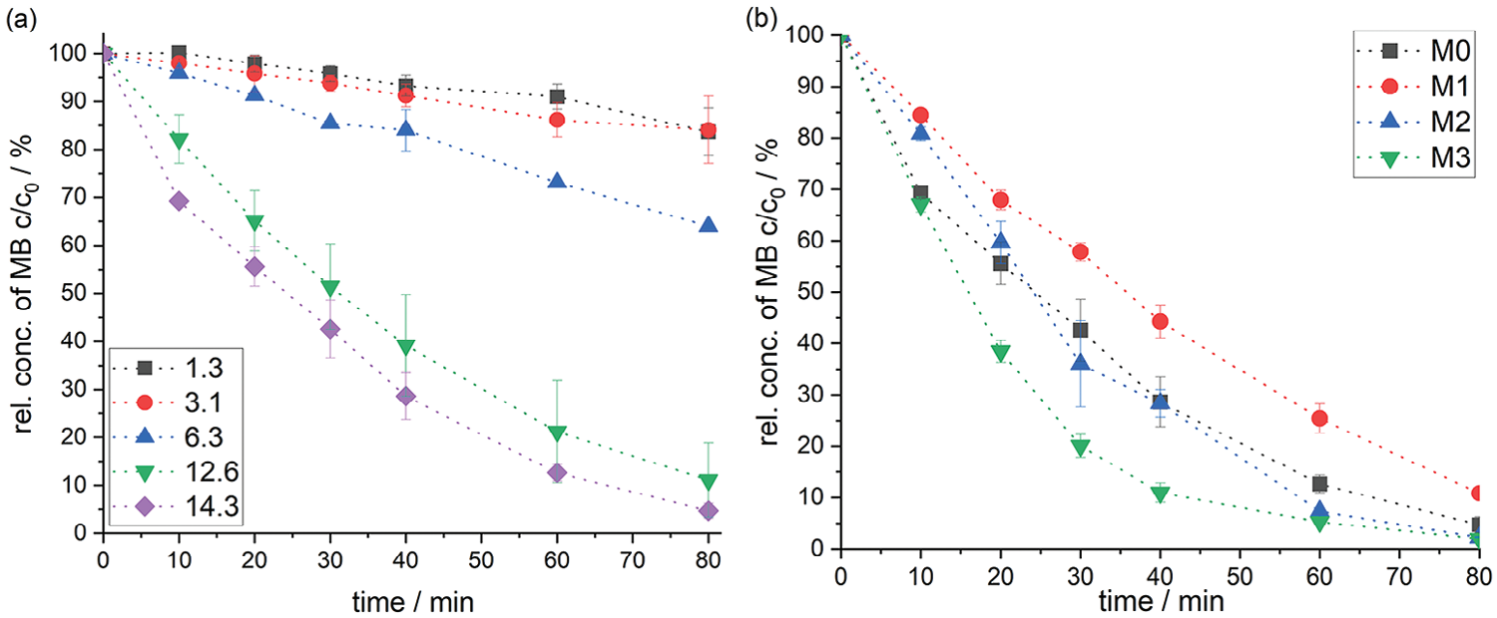
a) Degradation of methylene blue (MB) over time for Bi_2_WO_6_ applied on the PES membrane and synthesized at different concentrations of reactant salts (here stated as the concentration of Na_2_WO_4_ in g L^−1^). The reactant salts Na_2_WO_4_*H_2_O and Bi(NO_3_)_3_*5H_2_O were used at a ratio of 1:2.9, respectively. b) Degradation of methylene blue (MB) over time for Bi_2_WO_6_ applied by different methods on the PES membrane. M0: 90 s ultrasonic treatment, dip coating, washing, M1: 3 times 90 s ultrasonic treatment, dip coating, washing, M2: 90 s ultrasonic treatment, dip coating, drying, washing, M3: 90 s ultrasonic treatment, dip coating, drying, washing, second dip coating, drying, washing.

The degradation of methylene blue over time has been studied using Bi_2_WO_6_ PES membranes and is shown in **Figure** **3**a. The degradation of methylene blue after 80 min is only 16% for the PES membrane with Bi_2_WO_6_ particles synthesized with a low concentration of Na_2_WO_4_*2 H_2_O (1.3 and 3.1 g L^−1^). The highest degradation after 80 min with 95% removal was observed for the PES membrane with Bi_2_WO_6_ particles synthesized with a high concentration of Na_2_WO_4_*2 H_2_O (14.3 g L^−1^). Generally, the observed trend that with high crystallinity (high intensity and narrow peaks in the diffractogram) the photocatalytic activity increases is also evident here. The difference in degradation between PES membranes with Bi_2_WO_6_ particles synthesized at 12.6 and 14.3 g L^−1^ of Na_2_WO_4_*2 H_2_O is only small and partially within error values. Despite similar crystallinity, the PES membrane with Bi_2_WO_6_ particles synthesized with a concentration of 6.3 g L^−1^ of Na_2_WO_4_*2 H_2_O shows low photocatalytic activity here compared to Bi_2_WO_6_ particles synthesized with higher salt concentrations. But the amount of immobilized Bi_2_WO_6_ on the PES membrane differs also with the used reactant salt concentration of synthesis of Bi_2_WO_6_ (see Figure S1, Supporting Information). Therefore, a concentration of 14.3 g L^−1^ of Na_2_WO_4_*2 H_2_O during synthesis of Bi_2_WO_6_ is used for further investigations.

### 2.2 Influence of Immobilization Method

The immobilization method was adjusted to further increase the photocatalytic activity. The duration of ultrasonic treatment of Bi_2_WO_6_ particles before dip‐coating was varied and double dip‐coating was introduced with different drying and washing steps in between. Important for high photocatalytic activity is the amount of catalyst and its distribution on the PES membrane. The PES membrane immobilized with Bi_2_WO_6_ by method 3 (90 s ultrasonic treatment, dip coating, drying, washing, second dip coating, drying, washing) showed the highest amount of Bi on the PES membrane surface (see Figures S2, S3, and S4, Supporting Information). The amount of Bi has been quadrupled to (1.51 ± 0.47)%, thus degradation of methylene blue was increased to a removal of 98% after 80 min of irradiation (see Figure 3b).

### 2.3 Photocatalytic Activity

**Figure 4:**
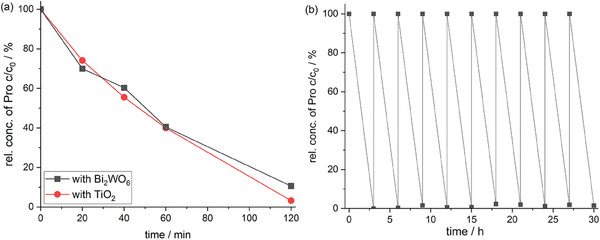
a) Degradation of propranolol (Pro) over time with the Bi_2_WO_6_ PVDF membrane and TiO_2_ PVDF membrane under irradiation with UVA light. TiO_2_ was hydrothermally synthesized.^[63]^ b) Degradation of propranolol (Pro) over time for 10 cycles (each 3 h) with the Bi_2_WO_6_ PVDF membrane under irradiation with UVA light. Bi_2_WO_6_ was synthesized for 24 h and with the concentration of the reactant salt Na_2_WO_4_*H_2_O adjusted to 14.3 g L^−1^ and immobilized with method 3 on the PVDF membrane.

The photocatalyst Bi_2_WO_6_ and TiO_2_ were immobilized on a PVDF membrane^[^ ^38^ ^]^ and the degradation of propranolol was compared (**Figure** **4**a). The change of the polymer membrane material from PES to PVDF was performed since the polymer PES is not stable under UVA light at longer irradiation times. The photocatalytic activity is only slightly decreased when Bi_2_WO_6_ is immobilized on a PVDF membrane compared to an immobilization on a PES membrane (see Figure S5, Supporting Information). Propranolol is a β‐adrenoceptors antagonist, which is used in the treatment of vascular diseases. More than 80% of propranolol is excreted via the urinary metabolites of humans. Unfortunately, propranolol is not significantly degraded in wastewater treatment plants, thus it is often found in surface waters.^[^ ^39^ ^]^ The degradation rate of propranolol with the TiO_2_ PVDF membrane is the same compared to the degradation rate with the Bi_2_WO_6_ PVDF membrane within the first 60 min of degradation. After 120 min of degradation, propranolol was degraded by 97% with the TiO_2_ PVDF membrane compared to 89% with the Bi_2_WO_6_ PVDF membrane.

To evaluate the stability and reusability of the Bi_2_WO_6_ polymer membrane, 10 additional cycles of propranolol removal were performed. Figure 4b shows the good recyclability of the Bi_2_WO_6_ polymer membrane for 10 consecutive cycles. The photocatalyst Bi_2_WO_6_ itself is not altering during 30 h of operation or is blocked by degradation products and is stably attached to the membrane, thus not lost during the 10 cycles.

The photocatalytic activity is influenced by the pH of the solution. In general, the pH has an impact on the adsorption of the molecule to be degraded due to the charge properties of the catalyst surface, the molecule to be degraded, and the photocatalyst itself. Zhou et al.,^[^ ^40^ ^]^ showed a shift of the conduction band energy (*E*_CB_) and the valence band energy (*E*_VB_) of Bi_2_WO_6_ from 0.51 to 0.33 eV and from 3.32 to 2.48, respectively when increasing the pH from 2.6 to 6.0. As with decreasing pH values, the E_VB_ becomes more positive, photooxidation activities are enhanced.^[^ ^40^ ^]^ Adsorption of propranolol to the TiO_2_ PVDF membrane is neglectable for pH 4 and low for pH 6 and 11 at 8.5% and 7.1%, respectively. Propranolol is a weak base with a pK_a_ of 9.5.^[^ ^41^ ^]^ Thus, the adsorption of propranolol is not the main impact factor influencing the degradation at different pH values here. Degradation of propranolol increased with enhanced pH values (Figure S6, Supporting Information). At pH 11, propranolol undergoes complete degradation within 60 minutes, whereas at pH 6 and pH 4, 91.5% and 84.7% of the substance are removed, respectively. The dependence of degradation activity regarding different pH values is controversial discussed in the literature due to different properties of the molecule to be degraded and apparently also the properties of Bi_2_WO_6_ itself. Bi_2_WO_6_ is described to be unstable in acidic solutions^[^ ^26^ ^]^ and under strongly alkaline conditions.^[^ ^40^ ^]^ An optimum degradation was found at around 7,^[^ ^26^, ^42^ ^]^ as well as with decreased^[^ ^40^, ^43^ ^]^ and increased pH.^[^ ^44^ ^]^ Adsorption of degradation transformation products at the PVDF^[^ ^45^ ^]^ membrane or Bi_2_WO_6_ which hinders further degradation can be enhanced at a certain pH, thus could also lead to an increase of overall removal of propranolol at varied pH values.

### 2.4 Visible Light Activity

The bandgap energy of Bi_2_WO_6_ is evaluated by analyzing the diffuse reflectance spectra and integrating the Kubelka–Munc function *F*(*R*_∞_) into the Tauc function by Davis and Mott (see for more details in the Experimental Section and Figure S7, Supporting Information) and is estimated to be 2.91 eV (426 nm). The bandgap of TiO_2_ was evaluated to be slightly higher with a value of 3.02 eV.^[^ ^19a^ ^]^

The calculated density of states for TiO_2_ and Bi_2_WO_6_ are shown in Figure S8 (Supporting Information). The calculated bandgap energies are 2.1 eV and 1.9 eV for TiO_2_ and Bi_2_WO_6_, respectively. As expected, the bandgap energies calculated by DFT underestimate the experimentally obtained values. Figure S9 (Supporting Information) shows the projected density of states for TiO_2_ and Bi_2_WO_6_. The valence and conduction band edges for Bi_2_WO_6_ are higher than those of TiO_2_. The valence band edge for both photocatalysts comprises mainly of O‐2p states, in the case of Bi_2_WO_6_, it also hybridized with Bi‐6s and Bi‐6p states to give the total density of states (DOS). The conduction band edge in TiO_2_ is comprised mainly of Ti‐3d states, while Bi_2_WO_6_ is comprised of the W‐5d mixed with O‐2p and Bi‐6p states. These results indicate that, in Bi_2_WO_6_, the charge transfer upon photoexcitation occurs from the O‐2p+Bi‐6s hybrid orbitals to the empty W‐5d orbitals. The visible light absorption of Bi_2_WO_6_ could be attributed to this transition from Bi‐6s to the W‐5d orbital. Furthermore, the hybridization of the Bi‐6s and O‐2p states makes the valence band largely dispersed, which favors the mobility of photogenerated holes and is beneficial to the oxidation reaction.^[^ ^46^ ^]^

**Figure 5:**
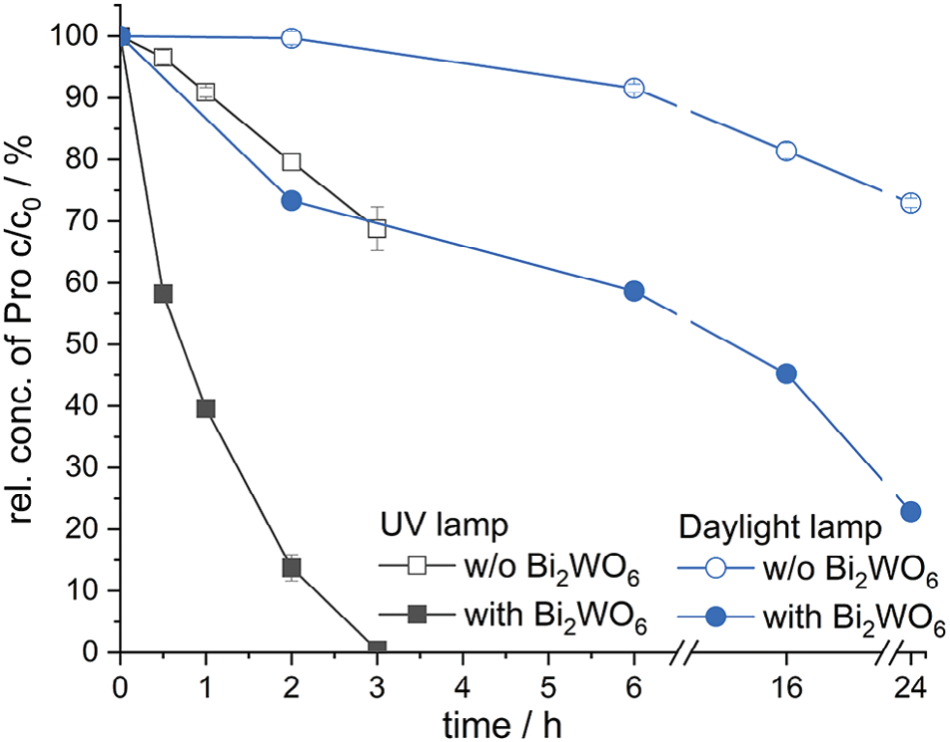
Degradation of propranolol (Pro) over time w/o the Bi_2_WO_6_ PES membrane (only photolysis) and with Bi_2_WO_6_ PES membrane (photocatalysis) under irradiation with UVA and daylight lamp. Bi_2_WO_6_ was synthesized for 24 h and with the concentration of the reactant salt Na_2_WO_4_*H_2_O adjusted to 14.3 g L^−1^ and immobilized with method 3 on the PES membrane.

The degradation in visible light with the Bi_2_WO_6_ PES membrane was proven by degrading propranolol with a daylight lamp. The degradation rate of propranolol is significantly slower under irradiation with visible light than with UVA light (**Figure** **5**). After 24 h of visible light irradiation, 73% of propranolol is degraded, complete degradation was not achieved in the time period tested. When irradiated with UVA light, degradation is completed after 3 h. The photolysis of propranolol is faster when irradiated with UVA light than with visible light. After 2 h of UVA light exposure, 20% of propranolol is photolyzed while visible light exposure resulted in no measurable change in concentration after 2 h. The energy of light in the visible light region is lower, thus less O_2_^•−^ radicals and holes h^+^ are generated.

### 2.5 Stability of the Polymer Membrane

The membrane stability under reactive oxygen species (ROS) attack generated by the photocatalyst Bi_2_WO_6_ and TiO_2_ (activated via UVA light) was tested by irradiating the membrane with Bi_2_WO_6_ and TiO_2_ immobilized in ultrapure water for 7 d (see experimental section for details).

**Figure 6:**
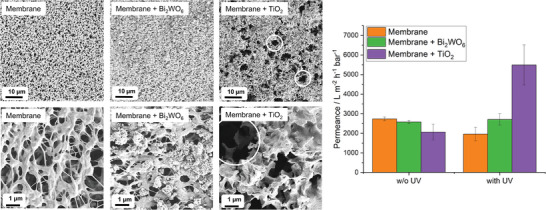
a) SEM images (two different magnifications) and b) water permeance of a PVDF membrane immobilized with Bi_2_WO_6_ and TiO_2_ and treated with UVA light in water for 7 d. In (a) several holes in the membrane morphology are marked with a white circle for membranes with immobilized TiO_2_. In (b) as a reference the untreated (w/o UV) permeance value is shown.

The PVDF membrane with and without Bi_2_WO_6_ is not degraded within 7 d of UVA irradiation (**Figure** **6**). The membrane morphology stays unchanged and the water permeance remains unaltered (PVDF membrane with Bi_2_WO_6_) or decreased marginally (pure PVDF membrane) due to an increase of the water contact angle from 77° to 103°. The pre‐hydrophilization of the membrane by the manufacturer is not stable under UVA irradiation. On the other hand, PVDF membranes with TiO_2_ immobilized show big holes (Figure 6), and the water permeance drastically increased from (2062 ± 405) LMH bar^−1^ to (5492 ± 1025) LMH bar^−1^ (Figure 6) due to ROS attack and destruction of the underlying PVDF polymer membrane.

**Figure 7:**
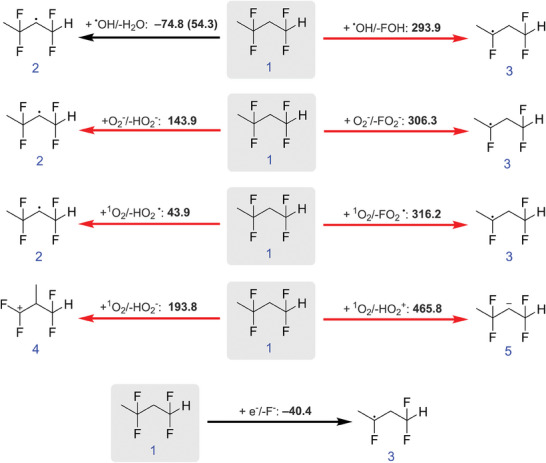
Calculated free reaction enthalpy Δ*G* in kJ mol^−1^ (M06‐2X/def2‐TZVP). Black arrows indicate possible exergonic reactions while red errors highlight endergonic reactions. The single value in a bracket is the activation barrier for an abstraction of a hydrogen atom by an OH^•^ radical from PVDF.

Reactions of PVDF with potential reactive species were investigated by density functional theory, see **Figure** **7**. Solely two reactions are exergonic, the abstraction of a hydrogen atom by an OH^•^ radical forming H_2_O and the reaction of a solvated electron with PVDF releasing a fluoride anion. Thus, O_2_^•−^ cannot react with PVDF. Additionally, the activation barrier for a hydrogen atom abstraction from PVDF by an OH^•^ radical is below 80 kJ mol^−1^. Thus, OH^•^ radicals can react with PVDF at room temperature. The activation barrier for the reaction of a solvated electron with PVDF could not be determined due to the limitations of static quantum chemistry approaches. Thus, the release of fluoride anions might be kinetically prohibited. TiO_2_ does form highly reactive OH^•^ radicals (see Table S1 and Figures S11 and S12, Supporting Information) which reacts with PVDF via abstraction of a hydrogen atom to form H_2_O. Instead, Bi_2_WO_6_ degrades organic molecules via O_2_^•−^ radicals or via direct oxidation by the hole h^+^, thus the polymer is not affected. No OH^•^ radicals have been detected when irradiating Bi_2_WO_6_ with UVA light (Table S1 and Figures S11 and S12, Supporting Information).

## 3 Conclusion

The photocatalyst Bi_2_WO_6_ was synthesized and immobilized on polymer microfiltration membranes to remove micropollutants from water.

With increasing salt concentration an enhancement of crystallinity was observed, and degradation of methylene blue was increased from 16% to 95% of degraded methylene blue after 80 min of degradation. The amount of immobilized Bi_2_WO_6_ is another important factor that influenced the degradation rate of methylene blue. The immobilization procedure was adapted by combining ultrasonic treatment of the Bi_2_WO_6_ suspension, double dip‐coating with drying the membrane in between. The amount of Bismuth on the membrane surface was increased from 0.4% to 1.5% which led to an advance from 57% to 80% of methylene blue degradation after 30 min.

The photocatalytic activity of the PVDF membrane with Bi_2_WO_6_ is comparable to a PVDF membrane with TiO_2_. After 120 min 90% of propranolol was degraded. Degradation efficiency was not altered during 10 cycles (30 h) of photocatalytic treatment of propranolol. The photocatalytic activity is increased with enhanced pH value, already after 60 min propranolol was fully degraded.

The bandgap energy was set at 2.91 eV (426 nm), thus Bi_2_WO_6_ is photocatalytic active in the visible light region. The Bi_2_WO_6_ PVDF membrane successfully degraded propranolol with visible and UVA light. After 24 h of visible light irradiation, 73% of propranolol is degraded, complete degradation was not achieved in the time period tested. When irradiated with UVA light, degradation is completed after 3 h.

The Bi_2_WO_6_ PVDF membrane was intact after long‐term exposure (7 days) to UVA light in ultra‐pure water in contrast to a PVDF membrane with immobilized TiO_2_. The water permeance of the TiO_2_ PVDF membrane increased from 2062 LMH bar^−1^ to 5492 LMH bar^−1^ after UVA light exposure, while the water permeance of the Bi_2_WO_6_ PVDF membrane did not change and stayed within the error values after UVA light irradiation. TiO_2_ does form highly reactive OH^•^ radicals when irradiated with UVA light that reacts with the PVDF membrane via hydrogen abstraction. On the other hand, Bi_2_WO_6_ forms only O_2_^•−^ radicals and holes h^+^, which do not react with the PVDF polymer in the same way. OH^•^ radicals can react with PVDF at room temperature (exergonic) while O_2_^•−^ cannot react with PVDF (endergonic).

The here presented synthesis of Bi_2_WO_6_ and its immobilization on polymer membranes is a promising alternative to the TiO_2_ polymer membrane due to its visible light activity and enhanced stability.

## 4 Experimental Section

### Materials and Chemicals

Two microfiltration membranes from Merck Millipore (Burlington, MA, USA), the Express PLUS membrane (polyethersulfone PES, 0.22 µm) and the Durapore membrane (polyvinylidene fluoride PVDF, 0.22 µm) are used for immobilization. Sodium tungstate dihydrate (Na_2_WO_4_*2 H_2_O, 99+%, ACS reagent), bismuth (III) nitrate pentahydrate (Bi(NO_3_)_3_*5 H_2_O, ACS reagent), 7‐Hydroxycoumarin (umbelliferone, 99%) and methylene blue (96+%) have been purchased from Acros Organics (Fisher Scientific, Waltham, MA, USA). Milli‐Q water was supplied by Milli‐Q Integral System (EMD Millipore, Billerica, MA, USA). Ethylene glycol (C_2_H_6_O_2,_ ≥99%, for synthesis) was purchased from Carl Roth GmbH + Co. KG (Karlsruhe, Germany). Ethanol (99%, 1% methyl ethyl ketone, and isopropanol) was acquired from BÜFA Chemikalien GmbH & Co. KG (Hude‐Altmoorhausen, Germany) and propranolol hydrochloride (99%) from Alfa Aesar (Ward Hill, MA, USA). Hydrochloric acid (1 N, Reag. Ph Eur, Reag. USP), coumarin (for synthesis) and sodium hydroxide solution (1 N, Reag. Ph Eur, Reag. USP) were obtained from Merck KGaA (Darmstadt, Germany). 5,5‐Dimethyl‐1‐Pyrrolin *N*‐Oxid (DMPO) (95%) was supplied from Apollo Scientific (Bredbury, UK). The synthesis of TiO_2_ is described elsewhere.^[^ ^19^, ^38^ ^]^ In summary, 4 mL of titanium(IV) isopropoxide (TTIP) was added to 80 mL of 0.1 m HCl, and the mixture was stirred for 15 min at room temperature. The resulting solution was then heated in a Teflon lined reactor (DAB‐3 with a TFMTM‐PTFE inset, Berghof Products + Instruments GmbH, Eningen unter Achalm, Germany) to 210 °C for 20 h. After cooling, the solution was dispersed using an ultrasonic probe at 40 W (Sonoplus, HD2200 Generator, KE76 probe, BANDELIN electronic GmbH & Co., KG, Berlin, Germany) for 90 s, resulting in the formation of a TiO_2_ nanoparticle suspension. The membrane was positioned into the suspension and subjected to shaking for 5 min. Subsequently, the membrane was washed with MilliQ water for 3 × 30 min and dried in air.

### Synthesis of Bi_2_WO_6_ and Immobilization

The synthesis route of Yang et al.^[^ ^47^ ^]^ was modified. Na_2_WO_4_*2 H_2_O and Bi(NO_3_)_3_*5 H_2_O were first solved each in 35 mL of ethylene glycol. The amount of Na_2_WO_4_*2 H_2_O and Bi(NO_3_)_3_*5 H_2_O was varied but the ratio was fixed to 1:2.9, respectively. Both solutions were mixed and treated in a Teflon lined reactor (DAB‐3 with a TFMTM‐PTFE inset, Berghof Products + Instruments GmbH, Eningen unter Achalm, Germany) at 160 °C for 24 h. The solution of Bi_2_WO_6_ particles in ethylene glycol solution was cooled before further treatment. Different immobilization processes were applied and are described in the following. M0 (standard): 90 s of ultrasonic treatment of the Bi_2_WO_6_ dispersion, dip‐coating, and washing; M1: 3 times 90 s ultrasonic treatment of the Bi_2_WO_6_ dispersion, dip‐coating, washing; M2: 90 s of ultrasonic treatment of the Bi_2_WO_6_ dispersion, dip‐coating, drying, washing; M3: 90 s of ultrasonic treatment of the Bi_2_WO_6_ dispersion, dip‐coating, drying, washing, dip‐coating, drying, washing. Dip‐coating was performed for 5 min while shaking. Washing was 5 min with Milli‐Q water, 5 min with ethanol, 5 min with Milli‐Q water, and 30 min with Milli‐Q water of the membrane. The samples were dried in the air. The ultrasonic probe did run at 40 W (Sonoplus, HD2200 Generator, KE76 probe, BANDELIN electronic GmbH & Co., KG, Berlin, Germany).

### Scanning Electron Microscopy (SEM)

SEM images were prepared with an Ultra 55 SEM (Carl Zeiss Microscopy GmbH, Oberkochen, Germany). Before SEM imaging, the membrane had to be sputtered with a 30 nm chromium layer (Leybold Z400 sputter system, Hanau, Germany).

### X‐Ray Photoelectron Spectroscopy (XPS)

XPS spectra were taken at the Axis Ultra with a monochromatic Al Kα cathode (Kratos Analytical Ltd., Manchester, UK). The X‐ray source power was set at 150 W and the pass energy at 40 eV.

### Powder X‐Ray Diffraction (XRD)

XRD was measured with a Rigaku Ultima IV X‐ray diffraction spectrometer (Tokyo, Japan) with Cu Kα radiation (40 kV, 40 mA, scanning speed: 1° min^−1^, step size: 0.02°). It was not possible to directly perform XRD measurements of the Bi_2_WO_6_ particles on the membranes as the signals are superimposed by noise caused by the polymer structure of the membrane. Therefore, the excessive Bi_2_WO_6_ powder in suspension was refined after synthesis and used for XRD.

### Photocatalytic Degradation of Methylene Blue

The photocatalytic degradation test was performed in a batch test using 6‐well plates. For this purpose, a circle with a diameter of *d* = 3.3 cm was cut from the membrane and an additional hole (*d* = 1 cm) was made in the center of this circle to obtain a ring. Six of these rings were placed into adjacent wells of the 6‐well plate. All 6 wells were filled with 4 mL of a 13 mg L^−1^ methylene blue solution. The methylene blue concentration was followed by measuring the absorption with an Infinite M200 multimode microplate reader (Tecan Group Ltd., Männedorf, Switzerland) at a wavelength of 660 nm. The zero value of the absorption was measured immediately. The samples were shaken on a radial shaker with 200 rpm without irradiation to measure the dark adsorption for 30 min. The absorption of the solution was measured in 10 min intervals. Subsequently, three membranes were removed from the 6 wells (photolysis reference) and the samples were irradiated with a UVA lamp (Heraeus Original Hanau Suncare tanning tube 21/25 slim, Hanau, Germany, 309400 nm, (11.3 ± 1.3) mW cm^−2^) to perform photocatalysis for 80 min. Absorbance was measured every 10 min for the first 40 min and every 20 min thereafter. The non‐catalytic photolysis was subtracted from the photocatalytic measurement.

### Photocatalytic Degradation of Propranolol

The photocatalytic degradation of propranolol was tested under a UVA lamp (Heraeus Original Hanau Suncare tanning tube 21/25 slim, Hanau, Germany, 309400 nm, (11.3 ± 1.3) mW cm^2^) and daylight lamp (Relassy LED lamp 1200 W). The determination of the propranolol concentration was performed via reversed‐phase high‐performance liquid chromatography HPLC (Dionex UltiMate 3000) with an UV–vis detector. The chromatograms were monitored and analyzed with Chromeleon software version 6.8 SR 14. The detection wavelength was set at 290 nm. The approach is similar to the photocatalytic degradation test of methylene blue. The membrane was cut into circles with a diameter of *d* = 3.3 cm. The circles were placed into plastic petri dishes with a diameter of *d* = 3.4 cm and filled with 4 mL of a 25 mg L^1^ propranolol solution. For experiments at different pH values, hydrochloric acid and hydrogen hydroxide were added to the propranolol solution to adjust the pH value to 4, 6, and 11. The petri dishes were shaken on a radial shaker with 200 rpm without irradiation to measure the dark adsorption for 30 min and afterward with irradiation to analyze the photocatalytic activity. The petri dishes were refilled with Milli‐Q water up to the mass at the start of the experiment to balance the evaporation before samples for HPLC were collected after 0.5, 1, 2, and 3 h (UVA lamp) and 2, 6, 16, and 24 h (daylight lamp).

For stability and reusability experiments, photocatalytic degradation was performed up to 10 times (cycles) under the UVA lamp. Samples for HPLC were collected after each cycle. The PVDF membranes with Bi_2_WO_6_ were washed 3 × 10 min with Milli‐Q water (and stored after 2–3 cycles per day overnight in water) before starting the next cycle.

### Water Permeance

Water permeance was calculated based on filtration experiments using a stainless steel pressure filter holder (16 249, Sartorius, Göttingen, Germany) for dead‐end filtration. An amount of 100 mL of deionized water was filtered through the membrane (active area: 4.9 cm^2^) at 2 bar, and the time of flow‐through was recorded. Pure water permeance J was calculated following Equation (2).

(2) $$J\;=\frac{V}{t\cdot A\cdot p}\;$$

*V* is the volume of water, *t* is the time of flow‐through, *A* is the active area, and *p* is the applied pressure.

### Bandgap Energy

UV–vis reflectance spectroscopy (Cary 5000, Agilent, USA) was performed to gain the bandgap of Bi_2_WO_6_. The spectrometer was equipped with an external diffuse reflectance accessory (DRA 2500, 150 mm diameter). Spectralon was used as a reflectance standard. The bandgap energy is evaluated by analyzing the diffuse reflectance spectra and integrating the Kubelka–Munc function *F*(*R*_∞_) from 1931^[^ ^48^ ^]^ into the Tauc function by Davis and Mott.^[^ ^49^ ^]^

(3) $$F\;\left(R_{\infty }\right)=\frac{K}{S}\;=\frac{{\left(1-R_{\infty }\right)}^{2}}{2R_{\infty }}\;$$

Here, $R_{\infty }=\frac{R_{\mathrm{sample}}}{R_{\mathrm{standard}}}\;$ is the reflectance of an infinitely thick sample. *K* and *S* are the absorption and scattering coefficients, respectively.^[^ ^50^ ^]^

(4) $${\left(\alpha \cdot h\upsilon \right)}^{1/\gamma }=\;B\left(h\upsilon -E_{g}\right)$$

Here, α is the absorption coefficient, *h* is the Planck constant, υ is the photon's frequency, *B* is the absorption constant, and *E*_g_ is the bandgap energy. The γ factor is based on the type of electron transition and is equal to 1/2 or 2 for the direct and indirect allowed transition bandgaps, respectively. α is replaced by *F*(*R*_∞_) to gain the following Equation (4).

(5) $${\left(F\left(R_{\infty }\right)\cdot h\upsilon \right)}^{1/\gamma }=\;B\left(h\upsilon -E_{g}\right)$$

With orthorhombic Bi_2_WO_6_ being an indirect semiconductor,^[^ ^51^ ^]^ ${\left(F(R_{\infty })\cdot h\upsilon \right)}^{1/2}$ can be plotted against hυ (see Figure 4a). The bandgap energy can be then evaluated from the intercept of the baseline and fitted line^[^ ^52^ ^]^ (see Figure S7, Supporting Information).

### Density of State Calculations

Density functional theory (DFT) calculations were carried out using the Quantum ESPRESSO code.^[^ ^53^ ^]^ Electron wave functions are expanded in the form of a plane‐wave basis set up to the cutoff energy 50 Ry, and the core‐valence interaction was treated using standard solid‐state pseudopotentials (SSSP PBE Efficiency v1.3.0).^[^ ^54^ ^]^ The electronic exchange and correlation were treated using the generalized gradient approximation (GGA), employing the Perdew–Burke–Ernzerhof (PBE) functional.^[^ ^55^ ^]^ A *k*‐points sampling under the Monkhorst–Pack^[^ ^56^ ^]^ scheme was used for the first Brillouin zone integration using a 9 × 9 × 7 and 6 × 6 × 2 *k*‐mesh in the geometry optimization and SCF calculations of TiO_2_ and Bi_2_WO_6_, respectively, and the Marzari–Vanderbilt^[^ ^57^ ^]^ smearing scheme with a Gaussian spreading of 0.02 Ry. Density of states (DOS) calculations were performed using a denser *k*‐points mesh of 18 × 18 × 14 and 12 × 12 × 4 for TiO_2_ and Bi_2_WO_6_, respectively. To achieve the minimum potential energy configuration of the system, the atomic positions and structure constants are optimized using the Broyden–Fletcher–Goldfarb–Shanno (BFGS) procedure. The DOS is calculated from the relaxed structures obtained by optimizing the orthorhombic Bi_2_WO_6_, crystallized in the *Pca2_1_* space group, and the tetragonal TiO_2_ (anatase), crystallized in *I4_1_/amd* space group. The DOS for TiO_2_ with brookite (orthorhombic *Pbca* space group) and rutile (tetragonal *P4_2_/mnm* space group) structure are also calculated and presented in Supporting Information (Figure S12).

### Long‐Time Stability Under UVA Light Irradiation

The stability of the membrane under long‐time (7 d) UVA light irradiation was investigated by placing the membrane in a glass petri dish filled with Milli‐Q water and irradiate it with the UVA lamp (Heraeus Original Hanau Suncare tanning tube 21/25 slim, 309–400 nm, (11.3 ± 1.3) mW cm^−2^) for 7 d. The surface of the membrane before and after irradiation was studied via SEM.

### Detection of OH^•^ Radicals—Reaction of Coumarin to Umbelliferone

Coumarin was utilized as a probe molecule to detect OH^•^ radicals, undergoing reactions that result in the formation of various OH‐substituted coumarins. Notably, only 7‐OH coumarin (umbelliferone) exhibits intense fluorescence, and the corresponding yields have been documented.^[^ ^58^ ^]^ To prevent possible adsorption of coumarin or umbelliferone to the polymer membrane only pure photocatalyst has been probed. Excessive Bi_2_WO_6_ and TiO_2_ powder in suspension were refined after synthesis.

The experimental procedure for the fluorescence probe method was as follows: 5 mg of photocatalyst powder was suspended in a plastic petri dish with a diameter of d  =  3.4 cm of 4 mL of a 0.1 mM coumarin aqueous solution. The petri dish was positioned under a UVA lamp (Heraeus Original Hanau Suncare tanning tube 21/25 slim, Hanau, Germany, 309–400 nm, (11.3 ± 1.3) mW cm^−2^) for 2 min. Following irradiation, the suspension was centrifuged at 12.000 rpm for 5 min (SIGMA 3–18K Centrifuge, Sigma Laborzentrifugen GmbH, Osterode, Germany), the clear solution was extracted, and its fluorescence spectrum was measured using a fluorescence spectrophotometer (Infinite M200 multimode microplate reader, Tecan Group Ltd., Männedorf, Switzerland) with an excitation wavelength at 332 nm and detection wavelength at 445 nm.

To convert fluorescence intensity into the concentration of umbelliferone, fluorescence spectra of various concentrations of umbelliferone in a 0.1 mM coumarin solution after dark adsorption with the photocatalyst were measured. An increase in fluorescence intensities at 445 nm demonstrated a proportional relationship with umbelliferone concentration up to 50 nM, as depicted in Figure S10 (Supporting Information). Utilizing this relationship, the concentration of umbelliferone generated in photocatalysis was calculated based on the fluorescence intensity of the solution. 6.1% of OH^•^ radicals react to umbelliferone when utilizing a 0.1 mM coumarin solution.^[^ ^58^ ^]^

### Detection of OH^•^ Radicals—Electron paramagnetic resonance (EPR)

Aqueous spin trap 5,5‐Dimethyl‐1‐Pyrrolin *N*‐Oxid (DMPO) solutions (100 mm) were irradiated for 60 min and transferred to a sample tube immediately after the irradiation experiment and analyzed by EPR spectroscopy within 15 min. Four different experiments of DMPO irradiation were performed, one each with TiO_2_‐modified and Bi_2_WO_6_‐modified PVDF membrane, one with unmodified PVDF membrane, and one with pure water. The EPR spectra were measured with an X‐band EPR spectrometer (ELEXSYS, BRUKER) with a cavity operating in the TE_102_ mode at room temperature. The spectrometer setup was: microwave power 2 mW, receiver gain 1–10^4^, modulation amplitude 0.05 mT. The simulation was performed using the software tool Easyspin.^[^ ^59^ ^]^

### Reaction of Reactive Compounds with PVDF

All calculations were carried out with the ORCA quantum chemistry program package.^[^ ^60^ ^]^ The M06‐2X functional was employed during the calculations since the large amount of Hartree–Fock exchange reduces errors from the self‐interaction error.^[^ ^61^ ^]^ Furthermore, M06‐2X was optimized to match the kinetic and thermodynamic properties of nonmetal compounds. Stationary points on the potential energy surfaces were confirmed by semi‐numerical frequency calculations. All calculations employed the revised triple basis set from the Ahlrichs group, def2‐TZVP.^[^ ^62^ ^]^ The solvation effects of water were considered by the Conductor‐like Polarizable Continuum Model (C‐PCM).^[^ ^63^ ^]^ Furthermore, the resolution of identity approximation^[^ ^64^ ^]^ and the chain‐of‐sphere^[^ ^65^ ^]^ approach were employed to accelerate all calculations. The experimental solvation enthalpy of the solvated electron of 151.9 kJ mol^−1^ was taken from the literature.^[^ ^66^ ^]^

## Conflict of Interest

The authors declare no conflict of interest.

## Supporting information

Supporting Information

## Data Availability

The data that support the findings of this study are available from the corresponding author upon reasonable request.
