# Supplementary material for: Immobilization of Bi2WO6 on Polymer Membranes for Photocatalytic Removal of Micropollutants from Water – A Stable and Visible Light Active Alternative
Source: Glob Chall. 2024 Feb 28;8(3):2300198. doi: 10.1002/gch2.202300198 (PMC10935888; doi:10.1002/gch2.202300198)
Supplement: Supplementary file 1 — Supporting Information [file GCH2-8-2300198-s001.pdf]

Global Challenges
Open Access

Supporting Information

for *Global Challenges.*, DOI 10.1002/gch2.202300198

Immobilization of Bi_2WO_6 on Polymer Membranes for Photocatalytic Removal of Micropollutants from Water – A Stable and Visible Light Active Alternative

Kristina Fischer, Amira Abdul Latif, Jan Griebel, Andrea Prager, Omid Shayestehpour, Stefan Zahn and Agnes Schulze*

Supporting Information

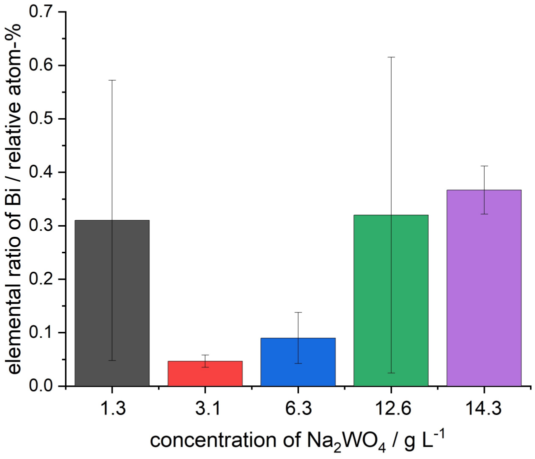

*Figure S1. Elemental ratio of Bi evaluated via XPS for Bi_2WO_6 applied on the PES membrane and synthesized at different concentrations of reactant salts (here stated as concentration of Na_2WO_4 in g L^{-1}). The reactant salts Na_2WO_4*H_2O and $Bi(NO_3)_3$*$5H_2O$ were used at a ratio of 1:2.9, respectively.*

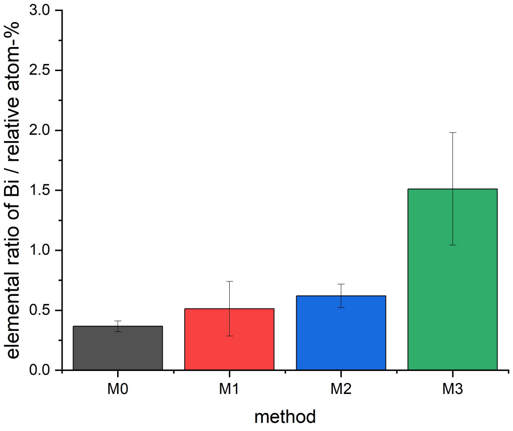

Figure S2 Elemental ratio of Bi evaluated via XPS for Bi_2WO_6 applied on the PES membrane by different methods. M0: 90 s ultrasonic treatment, dip coating, washing, M1: 3 times 90 s ultrasonic treatment, dip coating, washing, M2: 90 s ultrasonic treatment, dip coating, drying, washing, M3: 90 s ultrasonic treatment, dip coating, drying, washing, second dip coating, drying, washing.

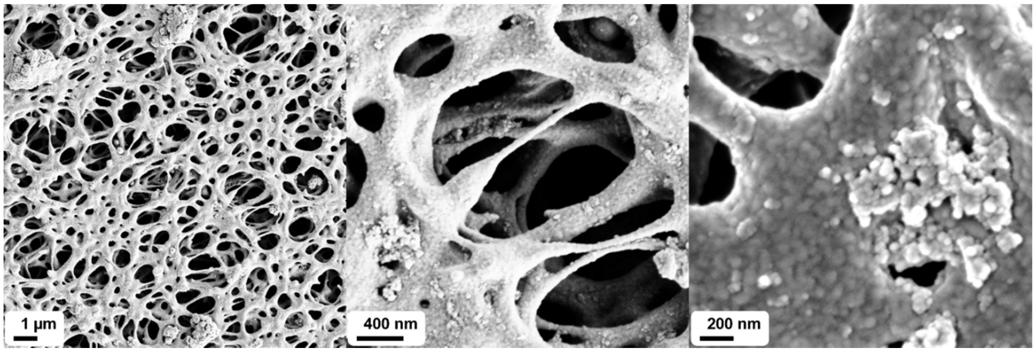

Figure S3. SEM images (different magnifications) of Bi_2WO_6 applied on a PES membrane (top view) with method 3 (concentration of $Na_2WO_4*H_2O$: 14.3 g L^{-1}, 24 h synthesis time, 90 sec of ultrasonic treatment, dip-coating, drying, washing, second dip-coating, drying, washing).

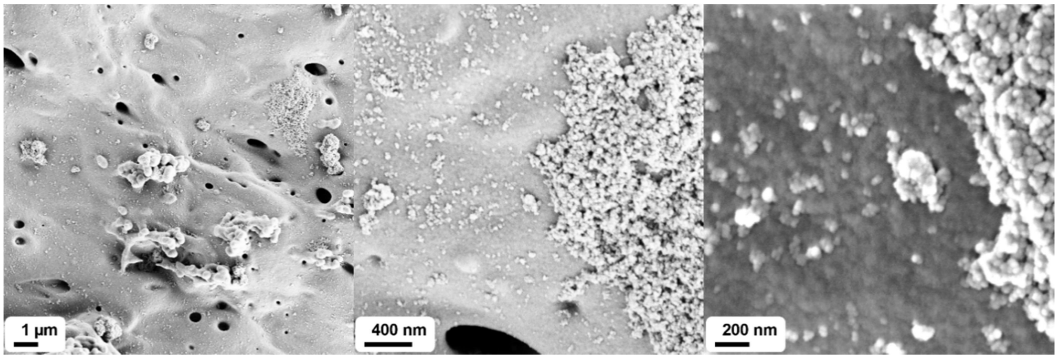

Figure S4. SEM images (different magnifications) of Bi_2WO_6 applied on a PES membrane (bottom view) with method 3 (concentration of $Na_2WO_4*H_2O$: 14.3 g L^{-1}, 24 h synthesis time, 90 sec of ultrasonic treatment, dip-coating, drying, washing, second dip-coating, drying, washing).

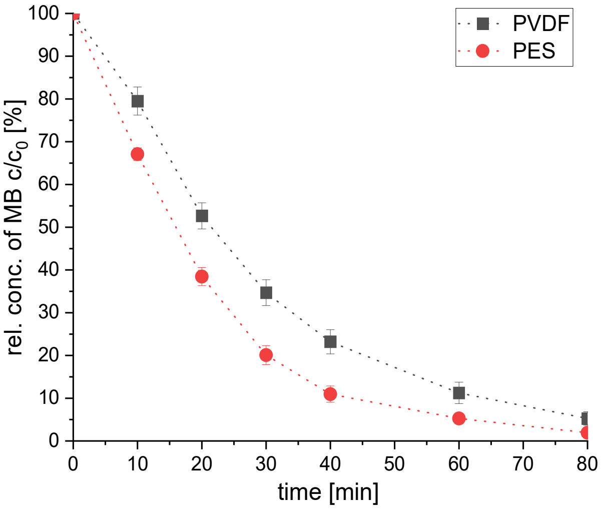

Figure S5. Degradation of methylene blue (MB) over time for Bi_2WO_6 applied on a PVDF and PES membrane with method 3 (concentration of $Na_2WO_4*H_2O$: 14.3 g L^{-1}, 24 h synthesis time, 90 sec of ultrasonic treatment, dip-coating, drying, washing, second dip-coating, drying, washing).

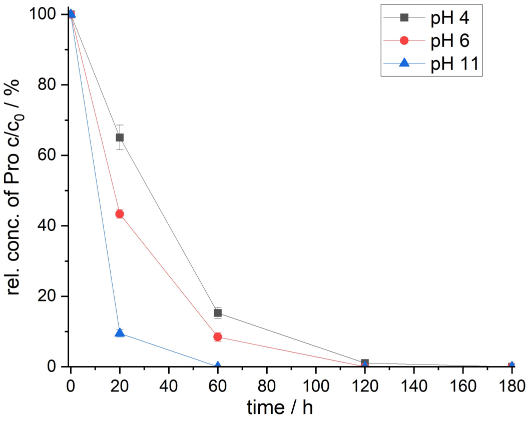

*Figure S6. Degradation of propranolol (Pro) over time at 3 different pH values with the Bi_2WO_6 PVDF membrane (photocatalysis) under irradiation with UVA- lamp. Bi_2WO_6 was synthesized for 24 h and with the concentration of the reactant salt Na_2WO_4*H2O adjusted to 14.3 g L^{-1} and immobilized with method 3 on the PVDF membrane.*

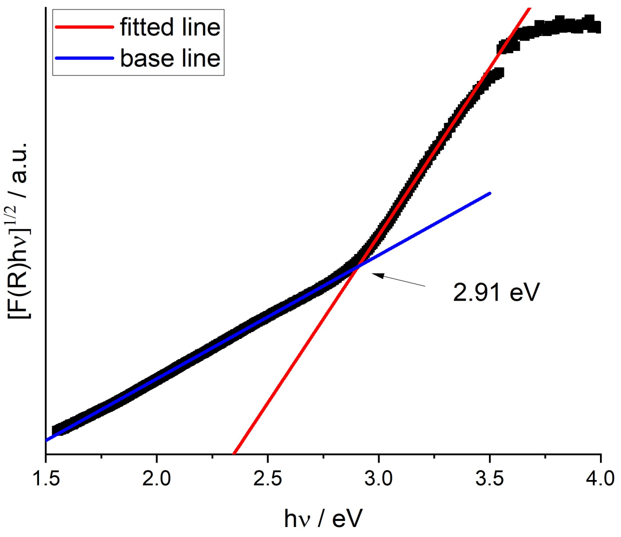

Figure S7. Plot of $(F(R_\infty)\cdot h\upsilon)^{1/2}$ as a function of the photon energy hυ.

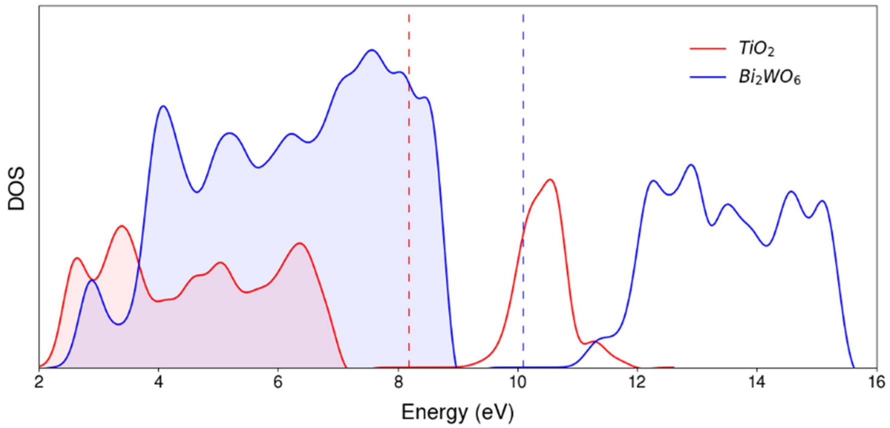

Figure S8. Calculated density of states of TiO_2 and Bi_2WO_6, and the corresponding Fermi levels (dashed lines) at 8.2 eV and 10.1 eV, respectively.

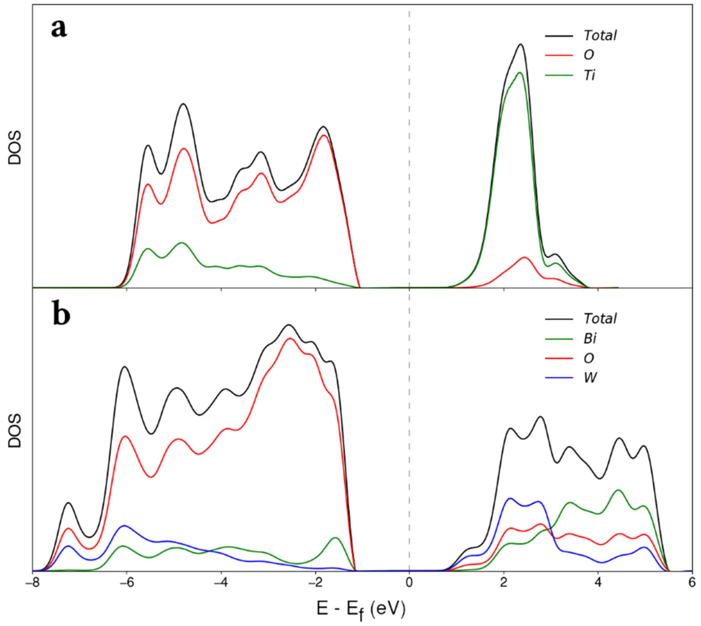

Figure S9. Projected density of states of TiO_2 (a) and Bi_2WO_6 (b), showing the elemental contributions to the total density of states.

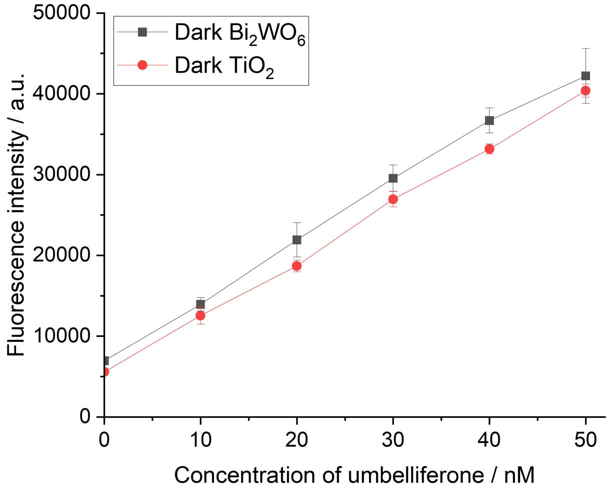

Figure S10. Fluorescence intensity in relationship to umbelliferone intensity.

Table S1. Fluorescence intensity after 2 min of irradiation and the responding concentration of umbelliferone using calibration of Figure S10. 6.1% of OH• radicals react to umbelliferone when utilizing a 0.1 mM coumarin solution[1].

Photocatalyst	Fluorescence intensity / a.u.	Concentration of umbelliferone / nM	Concentration of OH• radicals / nM
Bi_2WO_6	6386 ± 762	-0.75	0
TiO_2	24471 ± 2021 at 1:10 dilution	273.13	16.66

EPR measurement

Only the irradiated DMPO solution with TiO_2 membrane shows paramagnetic species. The other solutions are not paramagnetic and thus do not indicate the formation of OH• radicals. Figure S12 shows the EPR spectrum of the DMPO irradiated with the TiO_2 PVDF membrane together with its DMPO-OH adduct simulation. The isotropic EPR parameters are g_0=2.0057, $a_0(^{14}N)$=1.5 mT, and $a_0(^{1}H)$=1.5 mT. These values are in good agreement with the literature for the DMPO-OH adduct[2]. In addition, further hyper fine structure signals are observed. The assignment of these signals to a DMPO radical adduct could not be performed. The adduct DMPO-OOH can be excluded because the half-life of DMPO-OOH is approx. 80 seconds[3], and the irradiation experiment was not carried out directly in the EPR resonator. Even if not all signals could be assigned, the results of the EPR measurements prove that OH• radicals are generated during irradiation of the TiO_2 membrane and not with the Bi_2WO_6 membrane.

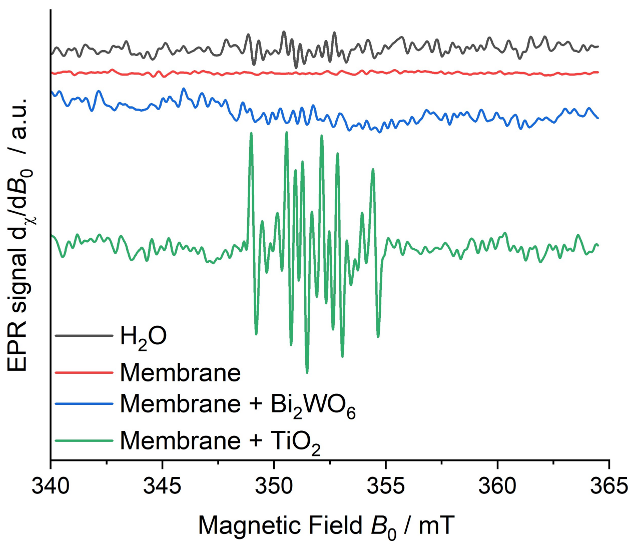

Figure S11. X band EPR spectra of aqueous reaction solution of DMPO irradiated (UVA) 15 min in advance for 60 min. Two reference samples were generated. Aqueous solutions of DMPO were irradiated - only water (H_2O) and with a PVDF membrane (Membrane). Membranes with immobilized photocatalysts (Membrane + Bi_2WO_6 and Membrane + TiO_2) in aqueous DMPO solutions were irradiated.

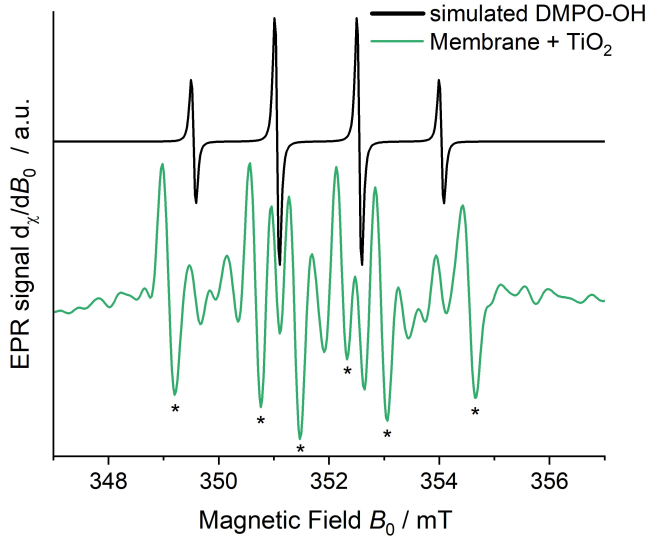

Figure S12. X band EPR spectrum of the aqueous reaction solution of the TiO_2 PVDF membrane with DMPO, and the simulation of an DMPO-OH adduct (simulated DMPO-OH). Additional signals of an unknown species are marked with asterisks.

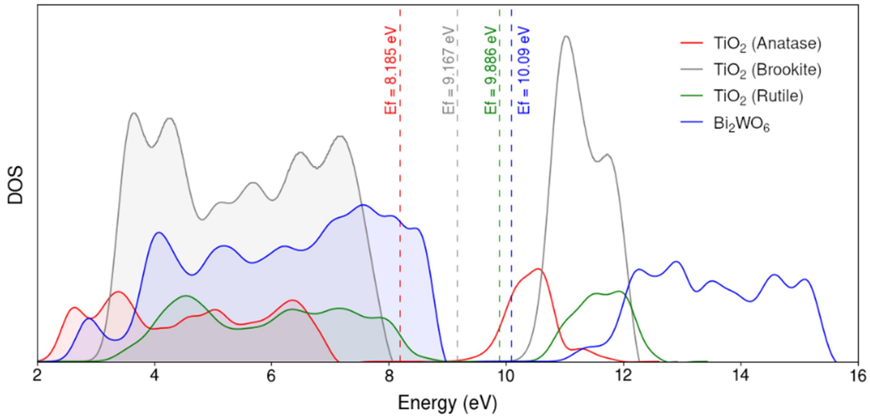

Figure S12. Calculated density of states for the three different crystal structures of TiO_2 are plotted alongside the density of states of Bi_2WO_6. Dashed lines are the corresponding Fermi levels.

Literature

[1] J. Zhang, Y. Nosaka, *The Journal of Physical Chemistry C* **2013**, *117*, 1383-1391.
[2] V. Brezová, Z. Barbieriková, D. Dvoranová, A. Staško, **2016**, *19*, 290-301.
[3] P. R. Marriott, M. J. Perkins, D. Griller, *Can. J. Chem.* **1980**, *58*, 803-807.
